# Identification of interchangeable cross-species function of *elongation factor-1 alpha* promoters in *Babesia bigemina* and *Babesia bovis*

**DOI:** 10.1186/s13071-016-1859-9

**Published:** 2016-11-11

**Authors:** Marta G. Silva, Donald P. Knowles, Carlos E. Suarez

**Affiliations:** 1 Department of Veterinary Microbiology and Pathology, Washington State University, Pullman, WA USA; 2 Animal Disease Research Unit, Agricultural Research Service, USDA, WSU, Pullman, WA USA

**Keywords:** *Babesia bovis*, *Babesia bigemina*, Transfection, Elongation factor-1α

## Abstract

**Background:**

Tick-borne *Babesia bigemina* is responsible for acute and potentially lethal hemolytic disease in cattle. The development of genetic manipulation tools necessary to the better understanding of parasite biology is currently limited by the lack of a complete parasite genome and experimental tools such as transfection. Effective promoters, required to regulate expression of transgenes, such as the *elongation factor-1 alpha* (*ef-1α*), have been identified in other apicomplexans such as *Babesia bovis* and *Plasmodium falciparum.*

**Methods:**

The *B. bigemina ef-1a* locus was defined by searching a partial genome library of *B. bigemina* (Sanger Institute). Presence of an intron in the 5’ untranslated region was determined by 5’ Rapid Amplification of cDNA Ends (RACE) analysis. Promoter activity was determined by measurement of luciferase expression at several time points after electroporation, efficiency of transfections and normalization of data was determined by quantitative PCR and by the percentage of parasitized erythrocytes.

**Results:**

The *ef-1α* locus contains two identical head to head *ef-1α* genes separated by a 1.425 kb intergenic (IG) region. Significant sequence divergence in the regions upstream of the inverted repeats on each side of the *B. bigemina* IG region suggest independent regulation mechanisms for controlling expression of each of the two *ef-1α* genes. Plasmid constructs containing the 5’ and 3’ halves of the IG regions controlling the expression of the luciferase gene containing a 3’ region of a *B. bigemina rap-1a* gene, were generated for the testing of luciferase activity in transiently transfected parasites. Both halves of the *ef-1α* IG region tested showed the ability to promote high level production of luciferase. Moreover, both *B. bigemina ef-1α* promoters are also active in transiently transfected *B. bovis* and conversely, a *B. bovis ef-1α* promoter is active in transiently transfected *B. bigemina.*

**Conclusions:**

Collectively these data demonstrate the existence of two distinct promoters with homologous and heterologous promoter function in *B. bigemina* and *B. bovis* which is described for the first time in *Babesia* species. This study is of significance for development of interspecies stable transfection systems for *B. bigemina* and for *B. bovis*.

**Electronic supplementary material:**

The online version of this article (doi:10.1186/s13071-016-1859-9) contains supplementary material, which is available to authorized users.

## Background

Tick and tick-borne diseases (TBDs) have vast impact on food production and public health. Bovine babesiosis is recognized among the most important TBD in terms of economic losses worldwide [1]. Bovine babesiosis can be in part controlled using live attenuated vaccines, but these vaccines are challenging to produce and distribute, have safety risks and a limited shelf life [2]. The existence of numerous research gaps concerning host-parasite relationships limit options for the development of improved methods for control. Novel research tools are needed in order to close such knowledge gaps and support development of new methods to control *Babesia* parasites and its vectors [3]. *Babesia bovis* and *B. bigemina* are the main parasites responsible for bovine babesiosis in terms of global parasite distribution, while *Babesia divergens* is mainly prevalent in the European continent [1].

In general *B. bovis* is regarded as the most virulent *Babesia* parasite, whereas, *B. bigemina* causes acute hemolytic disease [4]. Although the *B. bovis* genome and transfection systems were developed several years ago [5, 6], the genome for *B. bigemina* remains incomplete and unassembled (http://www.sanger.ac.uk/resources/downloads/protozoa/babesia-bigemina.html), and gene editing and transfection systems for *B. bigemina* remain unavailable. Attenuated transfected *Babesia* parasites are ideal candidates for delivery of anti-tick or other anti-TBD agents’ vaccine antigens [7]. Considering that *B. bigemina* has a wider range of tick vectors than *B. bovis*, including *Rhipicephalus* (*Boophilus*) *microplus* [8], *B. annulatus* [9] and *B. decoloratus* [10, 11] this parasite should also be considered as an efficient candidate for the development of dual parasite-vector vaccine approaches.

Gene editing and transfection systems, in addition to a complete genome, are required in order to advance our understanding of the molecular biology of *B. bigemina* parasites and for improved vaccine development. A current limitation for the development of such gene analysis systems is the lack of defined *B. bigemina* promoters and a method to introduce foreign DNA into *B. bigemina*. The promoters controlling the expression of elongation factor 1-alpha (*ef-1α*) genes in apicomplexan parasites are generally regarded as strong constitutive promoters [12, 13]. In *B. bovis* the *ef-1α* includes an arrangement of two identical head to head genes separated by a 1.4 kb region containing two apparently distinct promoters [12, 13]. Therefore a rational approach for the identification of *B. bigemina* promoters consists of searching for the presence of a similar structural and functional arrangement for the *ef-1α* locus in this parasite.

Given the current unavailability of a genetic system to better characterize the molecular basis for *B. bigemina* virulence and to facilitate vaccine development, we posed to define promoters and a method to introduce foreign DNA into the parasite. In this study we describe the *ef-1α* locus of *B. bigemina*, and tested the activity of *ef-1α* promoters. The data generated in this study may serve as the foundation for the future development of urgently needed gene editing and stable transfection systems for *B. bigemina*.

## Methods

### Parasites

The *B. bigemina* Puerto Rico strain [14] and *B. bovis* Texas S_74_T3Bo strain [15] were grown in long-term microaerophilous stationary-phase (MASP) culture using previously described methods [16, 17]. Parasites were obtained from 4 12.5 cm^3^-flask expansions of *B. bigemina* cultures containing approximately 25 % infected red blood cells (iRBC) as determined by microscopic counting of Diff-Quick-stained (Dade Behring, Deerfield, USA) slides. Infected red blood cells from the expansion were centrifuged for 10 min at 400× *g*. The cell pellet was suspended in 4 ml of Cytomix (120 mM KCl, 0.15 mM CaCl_2_, 10 mM K_2_HPO_4_/KH_2_PO_4_, 25 mM HEPES, 2 mM EGTA, 5 mM MgCl_2_, pH 7.6) and centrifuged again for 10 min at 400× *g*. The supernatant was removed and the tube of packed red blood cells was placed on ice until needed (Electroporation sub-section).

### Parasite DNA extraction

Genomic DNA (gDNA) was extracted from cultured *B. bigemina* Puerto Rico strain and cultured *B. bovis* Texas strain using the Qiagen Blood core kit (Valencia, USA) following the manufacturer’s instructions. The same kit was used to extract plasmid DNA (pDNA).

### Identification and sequencing of the *Babesia bigemina ef-1α* locus of the Puerto Rico strain

**Fig. 1:**
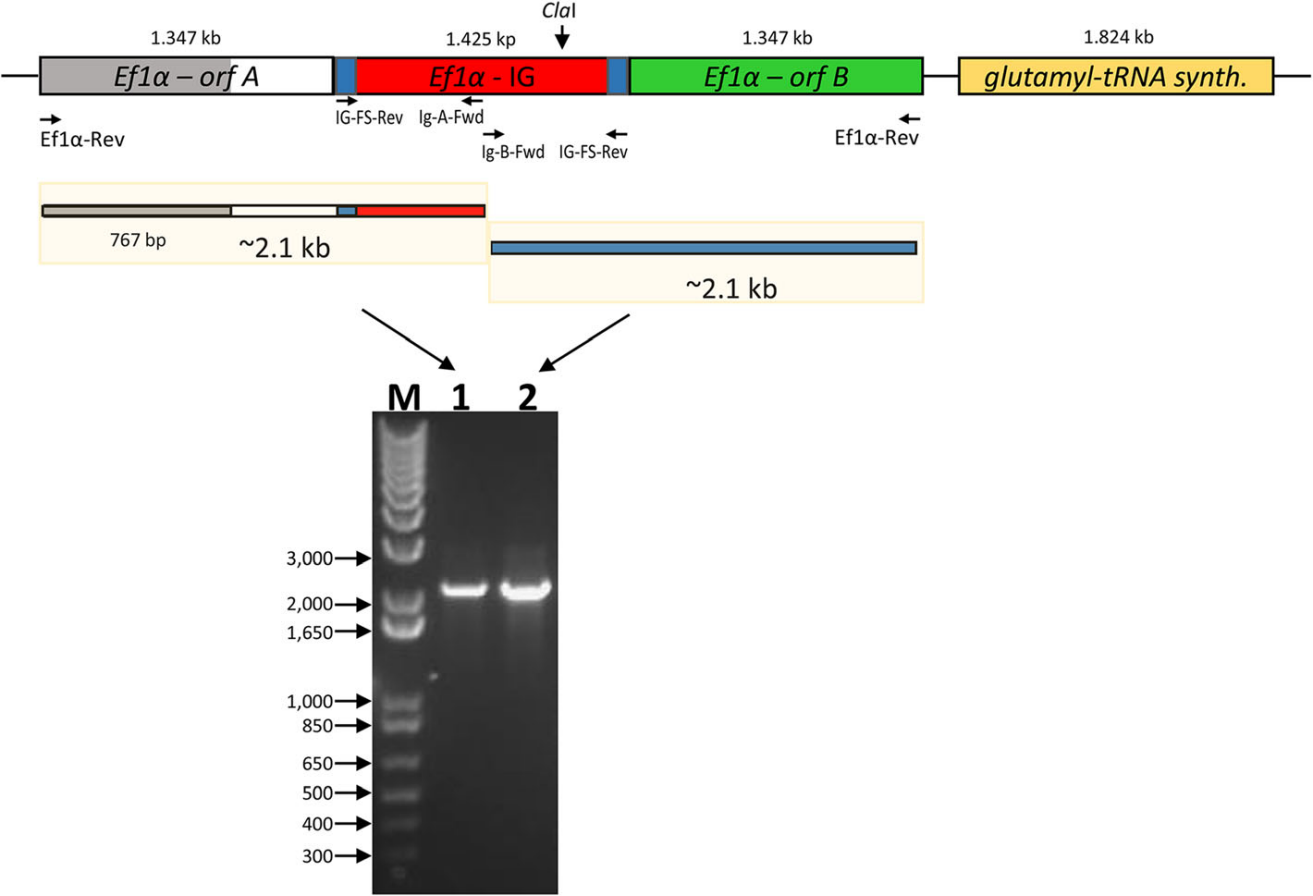
Definitive structure of the *B. bigemina ef-1α* locus*.* The structure of the *B. bigemina ef-1α* locus (upper panel) was deduced from the amplification of the PCR products obtained after amplification (lower panel). The previously 767 bp unknown sequence of the *ef-1α* locus is represented with a grey box. The location of primers used for the PCR amplification is marked with arrows. The regions on the IG containing IR repeats are represented by blue boxes, and the location of a *Cla*I site present in the IG region is also indicated with an arrow. PCR analysis performed on total *B. bigemina* gDNA amplicons on an agarose gel electrophoresis (lower panel). Lane 1, PCR product for Ig-A-Fwd + IG-FS-Rev primer; Lane 2, PCR product for Ig-B-Fwd + IG-FS-Rev. Molecular size marker is indicated on the left. *Abbreviation*: M, molecular size ladder in base pair (bp), 1000 bp DNA ladder

BLAST searches of the unfinished *B. bigemina* genome at the Sanger website (http://www.sanger.ac.uk/resources/downloads/protozoa/babesia-bigemina.html) were performed using the *B. bovis ef-1α* orf as a query (GenBank accession number: ALH43162.1). The full sequence of the *ef-1α* gene A in the *B. bigemina* Puerto Rico strain was determined by PCR analysis using the *ef-1α* orf reverse primer (Ef1α-Rev): 5′-CTT CTT GGA GGC CTT CTG GGC TG-3′ in conjunction with a primer present in the IG region, Ig-A-forward (Ig-A-Fwd): 5′-ATT AGG AAG CTT TAC AGA GGA CAT ACT TTA CTT G-3′ containing a Hind*III* restriction site at 5′ end. Primers Ig-A-Fwd and Ef1α-Rev and Ig-B-forward (Ig-B-Fwd): 5′-ATT AGG AAG CTT CAA GTA AAG TAT GTC CTC TG-3′ containing a Hind*III* restriction site at 5′ end and Ef1α-Rev were used to amplify both halves of the full *ef-1α* locus (Fig. 1). Both 2.075 and 2.063 kb amplicons were cloned into TOP-TA pCR2.1 cloning vector (Life technologies, Carlsbad, USA) and fully sequenced.

### Transfection plasmid constructs

*Babesia bigemina* gDNA was amplified using primers to amplify the Ig-A and Ig-B IG regions. For the Ig-A region a set of primers Ig-A-Fwd and IG-FS-reverse: 5′-CTT CTT GGA GGC CTT CTG GGC TG-3′, each containing a *Hind*III restriction site at the 5′ end, were used to amplify a 709 bp fragment. For the Ig-B region the set of primers Ig-B-Fwd and IG-FS reverse (sequence described above), each containing a *Hind*III restriction site at the 5′ end, were used to PCR amplify a 716 bp fragment. PCR products from both promoters were cloned into the TOP-TA pCR2.1 cloning vector (Life technologies) for sequence confirmation. Both Ig-A and Ig-B regions were then digested from the pCR2.1 plasmids with *Hind*III restriction enzyme (Promega, Madison, USA) and ligated into the linearized *Hind*III digested promoter-less “4-35-ef-luc” plasmid which contained the luciferase gene in the *EcoR*I site and the 3′ region of the *B. bigemina rap-1* in the *Pst*I site. All plasmids were sequenced to confirm that the promoters were in the correct orientation. A small 245 bp region of the 3′ region of *B. bigemina rap-1a* gene was amplified from gDNA of *B. bigemina* using the set of primers 3′ rap-a forward: 5′-GCG TCT CTG CAG TAA CAG CAA TTT AGC TGT AC-3′ and 3′ rap-a-reverse: 5′-CCG TAA CTG CAG ACA CGC TAT CTA CGG CCA TGG TGG C-3′, each containing a *Pst*I restriction site at the 5′ end. The *B. bovis* 3′ rap-1 region of the “4-35-ef-luc” plasmid was removed by digesting with *Pst*I restriction enzyme and replaced by ligation with the 245 base pair rap-1a 3′ region from the rap-1a gene of *B. bigemina* utilizing the same *Pst*I restriction site.

### RACE analysis: Analysis of *B. bigemina ef-1α* gene A transcripts

The *B. bigemina ef-1α* cDNA was specifically amplified using the SMARTer RACE cDNA Amplification Kit (Clontech, Mountain View, USA) protocol, the Advantage 2 PCR enzyme system and universal primer mix (UPM): 5′-CTA ATA CGA CTC ACT ATA GGG CAA GCA GTG GTA TCA ACG CAG AGT–3′. Primers race-1-ef-1α: 5′-CGT TCT TGG AGT CAG AAG CGA CGT GAC C-3′; race-2-ef-1α: 5′-GTA CGC TTG TCC ATA CGG CTG GTG ATC-3′; and race-3-ef-1α: 5′-CAG GGC TGG CAA CAT CGA TAA CTT CGT G-3′ were used for specific amplification of *B. bigemina ef-1α* gene A transcripts and amplified 5′ RACE products were cloned in vector 2.1-TOPO and sequenced.

### Electroporation

All electroporations were performed in a Gene Pulser II apparatus (BioRad, Richmod, USA) using 0.2-cm cuvettes. Twenty μg of plasmid was dried down in a Speedvac (Savant, Pittsburgh, USA) and suspended in 25 μl of Cytomix. Then, 37.5 μl of packed RBC were suspended in an equal volume of Cytomix, mixed with the plasmid briefly and transferred to a 0.2 cm cuvette follow electroporation. The conditions for electroporation were: 1.2 kV, 200 Ω and 25 μF. After electroporation, the cells were transferred to the well of a 48-well plate containing 5 % normal RBCs and HL-1 media with 40 % bovine serum to a final volume of 465 μl. The plate was then placed in the incubator until the luciferase assay was carried out. After electroporation, merozoites were cultured in vitro as described above.

### Luciferase assays

Luciferase analysis was performed as previously described in Suarez et al. [18]. Briefly, luciferase assays were performed using Promega’s LAR II detection reagent at room temperature and a Turner Designs TD-20/20 Tube Luminometer for a 10 s integration interval. For each set of luciferase assays, 2 μl of a 10^-6^ dilution of Promega’s QuantiLum Recombinant Luciferase diluted in 1× passive lysis buffer was assayed as a positive control. Half of the lysed sample material (25 μl) was used in the assays, and the remaining lysate was frozen at -20 °C for DNA extraction to be used in the Quantitative real-time PCR assay. Luciferase activity was measured at 4, 8, 24 and 48 h after electroporation in *B*. b*igemina* and *B. bovis* promoters, and at 72 h after electroporation for *B. bovis* promoter.

### Quantitative real-time PCR

A quantitative real-time PCR (qPCR) was standardized to assess the copy numbers of *luciferase* (luc) gene, *B. bigemina limulus coagulation factor C domain protein* (*ccp*)*-3* gene and *B. bovis merozoites surface protein* (*msa*)-1 gene in the transfected *B. bigemina* and *B. bovis* parasites. In *B. bigemina* culture samples were collected at 8 h post-electroporation, and in *B. bovis* culture samples were collected at 24 h post-electroporation. A set of primers were designed to amplify an: (i) 152 bp fragment of luc gene: 5′-GGT TTT GGA ATG TTT ACT-3′ and 5′-GCG AAG AAG GAG AAT AG-3′; (ii) 84 bp fragment of *B. bigemina* ccp-3 gene: 5′-TAC TCG TTC TAC ACA TTC C-3′, 5′-AAA CCA AGC AAC CTT AC-3′; and (iii) 150 bp fragment of *B. bovis* msa-1 gene: 5′-GAT GCG TTT GCA CAT GCT AAG-3′ and 5′- CGG GTA CTT CGG TGC TCT CA-3′. The qPCR were performed in a CFX96™ Real-Time PCR Detection System using the SsoAdvanced™ Universal SYBR® Green Supermix (Bio-Rad, Richmod, USA) for luc and ccp-3 genes and TaqMan® Universal PCR Master Mix (Life technologies, Carlsbad, USA) for *msa*-1 gene. The cycling conditions consisted of an enzyme activation step of 95 °C for 3 min followed by 40 cycles of 95 °C denaturation for 5 s and annealing/extension of 60 °C for 5 s for *luc* and *ccp-3* genes and for *msa-1* gene the cycling conditions consisted of an enzyme activation step of 95 °C for 10 min followed by 40 cycles of 95 °C denaturation for 10 s and annealing/extension of 55.8 °C for 15 s. Reactions were performed in triplicate in 20 μl volume using 500 nM of each primer for *luc* gene or 200 nM of each primer for *B. bigemina ccp-3* or *B. bovis msa-1* genes and 5 μl of pDNA used as standard curve and 2 μl of pDNA samples. The CFX Manager™ Software (Bio-Rad) was used to analyze the qPCR data, and the specificity and analytical sensitivity was assessed by melt curve analyses and standard curve, respectively. Copy numbers of *luciferase*, *B. bigemina ccp-3* and *B. bovis msa-1* genes were calculated based on a standard curve as previously described [19]. Statistical significance was analyzed using a two-sample *t*-test for differences among the treatment groups.

## Results and discussion

### Features and full sequence of the *ef-1α* locus in *B. bigemina*

The search for the *B. bigemina* Bond strain *ef-1α* locus identified a single contig (contig 4117.1) containing a truncated *ef-1α* orf followed by a 1.4 kb intergenic (IG) region and a full size *ef-1α* orf (Fig. 1). These data suggests that the *B. bigemina* genome contains an *ef-1α* locus which is structurally similar to the locus found in *B. bovis* and *Plasmodium* [12]. A full and identical *ef-1α* gene copy A was found to be located in the 5′ region upstream of the incomplete sequence reported in the Sanger database in the Puerto Rico *B. bigemina* strain. Further sequence analysis of the *ef-1α* locus of the Puerto Rico *B. bigemina* strain showed a putative glutamyl tRNA synthase gene located immediately upstream to the *ef-1α-B* gene (GenBank accession number XM_012911268.1). Synteny among these two genes is also conserved in the *B. bovis* and *Plasmodium* genomes [12]. Based on this information, we also obtained the full sequence of the *ef-1α* gene A and confirmed the full *B. bigemina ef-1α* locus structure by performing PCR amplifications on the Puerto Rico strain of *B. bigemina* (Fig. 1).

Full sequencing of the PCR products (Lane 1, Fig. 1) revealed an ef-1α orf A which is identical to the *ef-1α B* gene (Lane 2, Fig. 1), and provided novel sequence data for the IG region and *ef-1α* gene B, with a few base discrepancies when compared to data of the unfinished genome (GenBank accession number KT439182). Overall, analysis of the PCR products confirmed that *B. bigemina* contains an *ef-1α* locus which is identical in structure to the *B. bovis* locus, consisting of two identical 1.347 kb *ef-1α* orf’s which are separated by a 1.425 kb IG region as represented in the top panel of Fig. 1. Furthermore, BLAST searches performed with the full size *ef-1α* locus deduced from the Puerto Rico strain (GenBank accession number KT439182) shows 99 % identity with a 4.119 kb region located in the assembled Chromosome 1 of *B. bigemina* Bond strain (bp 1849423-1853541) (GenBank accession number LK391707).

**Fig. 2:**
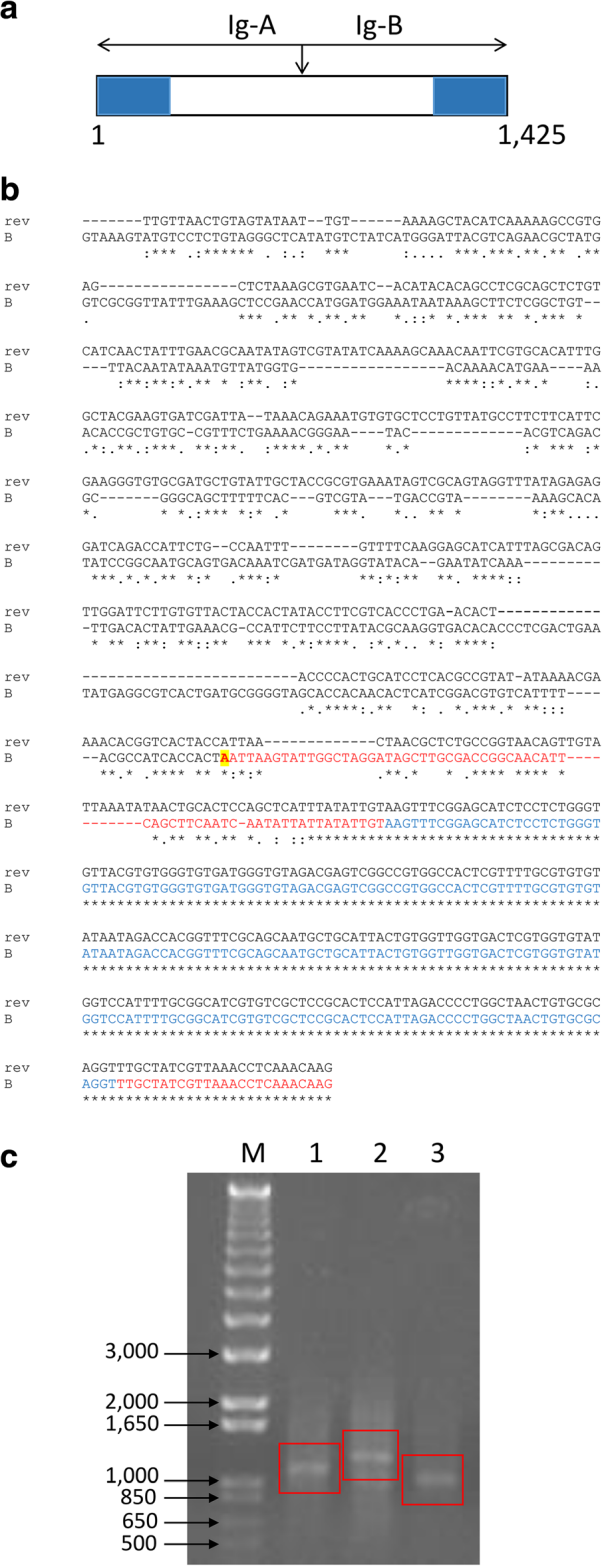
**a** Schematic representation of the *B. bigemina ef-1α* IG region. The full *B. bigemina ef-1α* IG region is represented in panel A, the regions containing the 243 bp IR are represented in blue boxes. The Ig-A (712 bp) and Ig-B (713 bp) fragments used for promoter analysis are represented with blue arrows. **b** Sequence of the 5’ end of the *ef-1α* transcript. Alignment of the Ig-A (rev) and Ig-B fragments of the IG region represented in Panel A. The 5’ end of the sequence of the RACE product is marked in red, and the intron in blue fonts. **c** Agarose gel electrophoresis analysis of the 5’ RACE products obtained from total *B. bigemina* RNA. Lane 1, PCR product for 5’- race-1-*ef-1α* + UPM primer; Lane 2, PCR product for race-2-*ef-1α* + UPM primer; Lane 3, PCR product for race-3-*ef-1α* + UPM primer. Amplicons are marked with a red box. Molecular size marker is indicated on the left. *Abbreviation*: M, molecular size ladder, 1000 bp DNA ladder

While the sequences of the *ef-1α* orf’s from *B. bovis* and *B. bigemina* are highly similar (88 % identity), the sequence of the *B. bigemina* IG region is unrelated (no significant homology detected) to the *B. bovis* IG region. Yet, the *B. bigemina* and the *B. bovis ef-1α* IG regions have similar structural features, including the presence of inverted repeats (IR) (Figs. 1 and 2a). The *B. bovis* IG region IR includes the first 260 bp in their 3′ and 5′ ends, whereas, the *B. bigemina* IG region has slightly shorter 243 bp IR regions. Oddly, a conserved *Cla*I site is located in exactly the same position in the *ef-1α* IG regions in both parasites (Figs. 1 and 2a) [12]. No other repetitive sequence was found in the *B. bigemina* IG region beyond the inverted repeat regions (Fig. 2a, b). Moreover, transcriptional analysis performed by RACE (Fig. 2) also demonstrated a putative start of transcription of the *ef-1α* and the presence of an intron in the 5’ UTR regions of the *B. bigemina* (Fig. 2b, c)*.* The sequence of the 5’-RACE-derived *ef-1α* transcript was deposited in GenBank (Accession number: KT439182). The presence of an intron in the 5’ UTR of the *ef-1α* orfs is another structural feature shared among the *B. bovis* and the *B. bigemina ef-1α* loci. The sequence and structure of the 5’UTR and the intron are shown in Fig. 2b.

### Homologous promoter activity of two distinct IG fragments demonstrated the presence of two distinct promoter regions

**Fig. 3:**
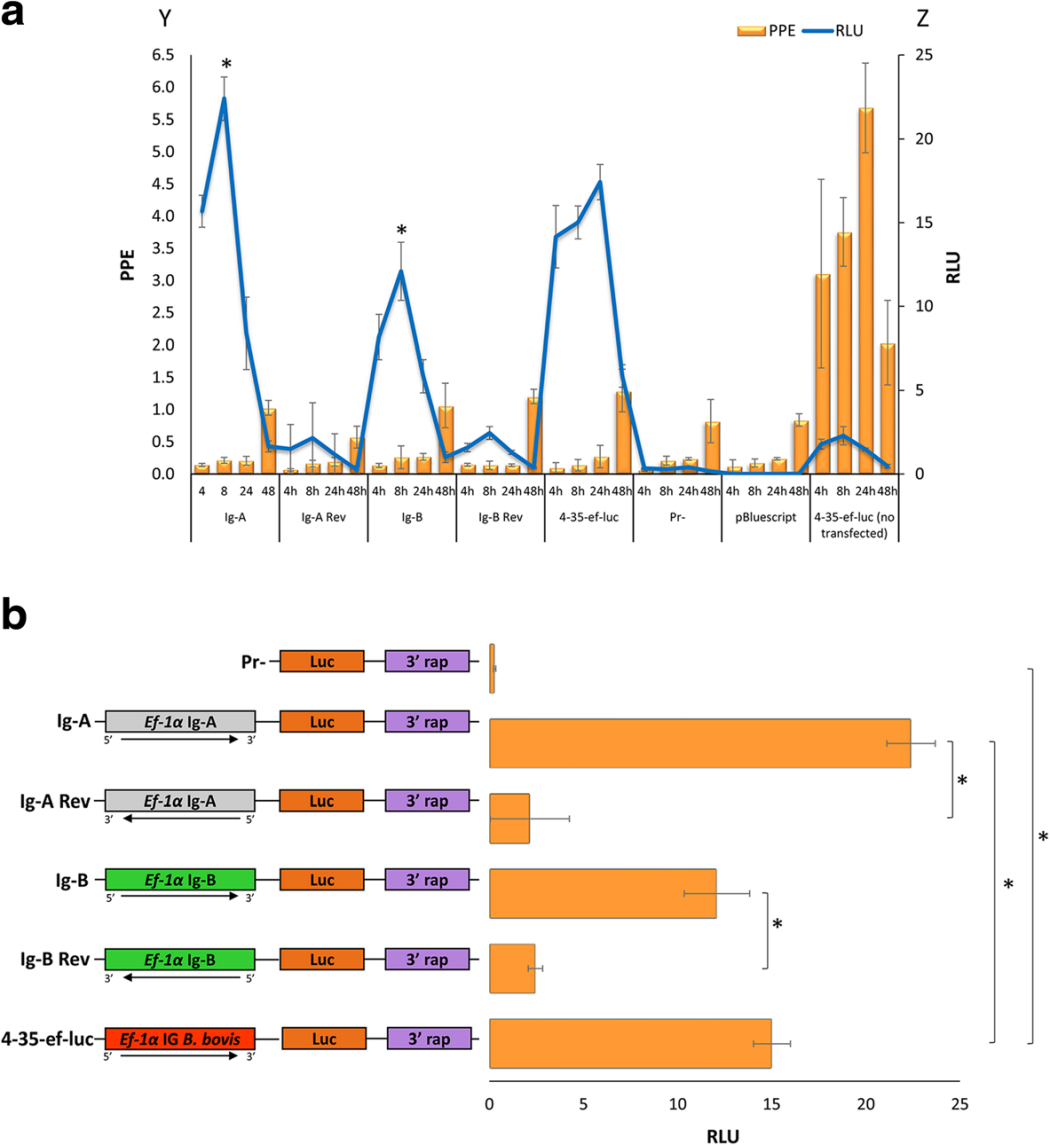
**a** Homologous function of the *B. bigemina* IG promoters. Functional promoter analysis of the *B. bigemina* IG region in *B. bigemina* transfected parasites. The Y axis represents the percentage of parasitized erythrocytes (PPE), and the Z axis represents Relative Luciferase Units (RLU). The X axis represents the different plasmid constructs (Ig-A, Ig-A Rev, Ig-B, Ig-B Rev, 4-35-ef-luc, Pr-, pBluescript) transfected and the control (4-35-ef-luc not transfected) at the time of the measurements performed at different hours post-electroporation. The black bars represent the PPE ± standard deviation (SD). The grey line represents relative luciferase units (RLU). **b** Schematic representation of the plasmid constructs used in the transient transfections and the RLU obtained eight hours after electroporation. Asterisks represent statistically significant differences detected using the t- test (*P* < 0.01)

**Fig. 4:**
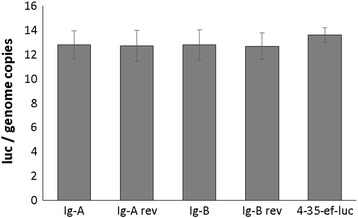
Efficiency of transient electroporation of *B. bigemina*. The number of plasmid DNA copies per genome was calculated from real time PCR analysis at eight hours after electroporation. The Y axis represents the ratio of plasmid/genome copies (Luc/genome copies) and the X axis represents the different plasmid constructs used in the electroporation. No significant differences were detected among the different plasmids electroporated into *B. bigemina*

The promoter activity of two fragments termed Ig-A and Ig-B, derived from each half of the *ef-1α* IG region of *B. bigemina* was tested using luciferase assays*.* The transient transfection plasmid contained either the fragment Ig-A representing 712 bp upstream of the *ef-1α* orf A, or the fragment Ig-B representing 713 bp upstream of the start of the ef-1α orf B (Fig. 2a). The plasmid vectors using in the transfections are shown in Fig. 3a and b. Luciferase expression reached a peak at 8 h after electroporation, regardless of the IG region used as promoter (Fig. 3a). The results comparing expression of luciferase at 8 h post-electroporation for each construct are shown in detail in Fig. 3b (*F*_(5,12)_ = 20.63, *P* = 0.000). Comparative growth curves and statistical analysis (Fig. 3a and Additional file 1: Figure S1a) shows that *B. bigemina* viability at 8 h post-electroporation was equally affected among all transfected plasmids regardless of the plasmid used for electroporation (*F*_(6,14)_ = 0.75, *P* = 0.617). In addition qPCR demonstrated similar efficiencies of transient transfection for all plasmid/parasite combination used in the study. The qPCR was performed using the *B. bovis* 3′ termination *msa-1*sequence, which is unique to the transfection plasmid, *ccp3*, a single gene copy of the *B. bigemina* genome and the *luc* gene [20] (Fig. 4). No significant differences in the number of plasmids per genome were found among the distinctly transfected parasites indicating that the ratios of plasmid gene copies per genome were identical for all plasmids electroporated in this experiment (Fig. 4) (*F*_(4,10)_ = 0.73, *P* = 0.589). This finding enables a comparison of the relative promoter strengths of the Ig-A and Ig-B regions used in the study. It can thus be concluded from these data that the Ig-B fragment, representing sequence upstream of the *ef-1α* orf-B in the *ef-1α* locus, is able to promote the highest level of luciferase activity when placed in the 5′ → 3′ orientation (*F*_(3,8)_ = 43.57, *P* = 0.0001 at 8 h), indicating that this fragment contains a relatively strong promoter. The Ig-A fragment cloned in the 5′ → 3′ orientation also promoted the expression of significantly higher luciferase activities compared to the control where the orientation of the sequence was reversed (*F*_(3,8)_ = 18.33, *P* = 0.001 at 8 h), and thus it also contains a promoter. Therefore, significant levels of luciferase expression were consistently obtained from transfected parasites containing the Ig-A and Ig-B fragments when cloned in the correct 5′ → 3′ orientation suggesting that the *ef-1α* IG region contains two independent promoters. The data indicates that the “Ig-B” region possesses higher promoter activity than the “Ig-A” region. However, this finding should not be interpreted as an indication of the actual strength of the “native” *ef-1α-A* and *ef-1α-B* promoters as they are arranged in the locus. True promoter strength should be estimated using full size IG regions cloned in the appropriate configuration relative to the target orf. Even if this is the case, this experimental approach, based on promoter regions cloned in transiently transfected plasmids, would not allow estimating the possible regulatory role of distantly located or “trans” enhancers, as well as the activity of other regulatory elements such as epigenetic factors. In addition, it is also possible that the promoter activity can be distinctly regulated in different stages of the life-cycle of the parasite, and the data discussed here may be applicable only to intra erythrocyte parasites developing in in vitro cultures. Therefore, it is likely that the absolute strength of these promoters as well as the study of its regulations could not be properly assessed using a limited transient transfection approach such as the one used in this study. In summary, both regions tested seem to have different promoter strengths, despite conservation of the inverted repeats, but sequence divergence upstream these regions may account for differences in promoter strengths. Despite of these considerations, the data allows for the selection of Ig-B as a relatively strong promoter to be used in future stable transfection experiments using in vitro cultivated parasites.

### Heterologous promoter activities of the *B. bigemina* and *B. bovis ef-1a* promoters

**Fig. 5:**
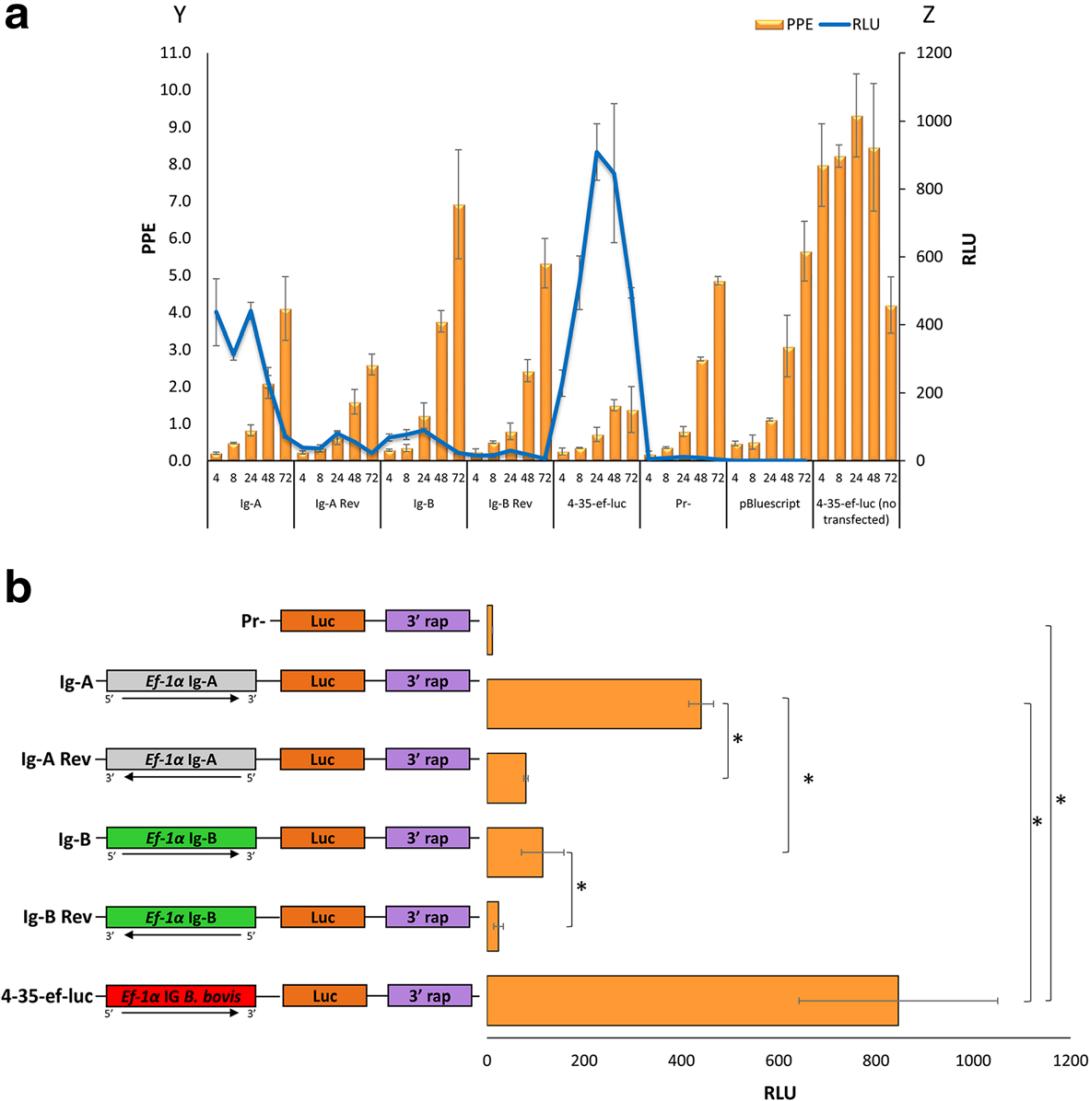
**a** Heterologous function of the *B. bigemina* IG promoters. Functional promoter analysis of the *B. bigemina* IG regions in *B. bovis* transfected parasites. The Y axis represents the PPE, and the Z axis represents RLU. The X axis represents the different plasmid constructs (Ig-A, Ig-A Rev, Ig-B, Ig-B Rev, 4-35-ef-luc, Pr-, pBluescript) transfected and the control (4-35-ef-luc not transfected) at the time of the measurements performed at different hours post-electroporation. The black bars represent the PPE ± standard deviation (SD). The grey line represents relative luciferase units (RLU). **b** Schematic representation of the plasmid constructs used in the transient transfections and the RLU obtained 24 h after electroporation. Asterisks represent statistically significant differences detected using the t- test (*P* < 0.05)

**Fig. 6:**
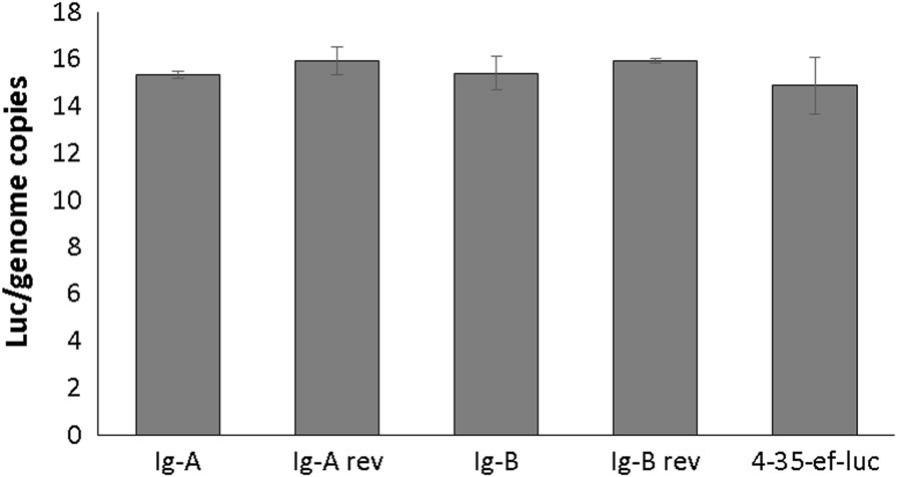
Efficiency of transient electroporation of *B. bovis* parasites. The number of plasmid DNA copies per genome was calculated from real time PCR analysis at twenty-four hours after electroporation. The Y axis represents the ratio of plasmid/genome copies (Luc/genome copies) and the X axis represents the different plasmid constructs used in the electroporation. No significant differences were detected among the different plasmids electroporated into *B. bigemina*

We then tested whether a promoter previously identified in the *ef-1α* IG region of *B. bovis* is also functional in *B. bigemina* and conversely, whether the *B. bigemina ef-1α* promoters described above are also functional in *B. bovis*. Luciferase activities in *B. bovis* transfected with plasmid 4–35 [5], containing the *B. bovis ef-1α* promoter for the *ef-1α B* gene (Fig. 5a) peaked at 24 h in all cases (Fig. 5a). After electroporation of the plasmids containing the *B. bigemina ef-1α* A and B promoters respectively into *B. bovis* parasites and, consistent with the data shown in Fig. 5a, we measured luciferase activities at its 24 h peak (Fig. 5b) (*F*_(5,12)_ = 12.03, *P* = 0.0001). The results of the comparative luciferase activities are shown in Fig. 5b. Analysis of growth rates performed at 8 and 24 h post-electroporation showed undistinguishable growth rates for all *B. bovis* transfected parasites (*F*_(6,14)_ = 0.92, *P* = 0.510 at 8 h and *F*_(6,14)_ = 2.07, *P* = 0.123 at 24 h), suggesting that parasite viability is equally affected by the procedure regardless of the transfection plasmid used in the electroporation (Additional file 1; Figure S1b). In addition, qPCR performed as described above demonstrates identical efficiencies for transfection regardless of the plasmid used (Fig. 6) (*F*_(4,10)_ = 1.27, *P* = 0.345), thus allowing comparisons of luciferase activities among the distinct promoters and estimating their relative strength under the conditions tested.

The *B. bovis ef-1α* promoter in Ig-B is able to promote expression of the luciferase gene in *B. bigemina* parasites at relatively high levels. This can be due to higher transfection efficiencies for the transference of the plasmid into *B. bigemina*, to higher affinity of the *B. bovis* promoter for *B. bigemina* transcription factors, or alternatively, the lack of proper regulatory signals that can be involved in the regulation of the activity of the *ef-1α* promoter in *B. bigemina*. However, data in Fig. 6 demonstrates that all plasmids were transferred into the parasite genome with identical efficiencies, thus precluding the first possibility. Regardless of the possible explanations for the observed increased efficiency, *B. bovis* promoter is functional in *B. bigemina* parasites. Conversely, the *B. bigemina* promoter is also functional in *B. bovis* parasites. Recent work performed in *B. bovis* suggested the need for the use of heterologous promoters to increase the efficiency of the targeting of transfected genes by homologous recombination in this parasite [21]. Data obtained in this study confirms the feasibility for the use of heterologous promoters that can be functional among these two parasites. This will allow the improvement of *B. bovis* transfection-based knockout (KO) and gene editing technologies, and the development of currently unavailable transfection KO systems for the functional gene characterization in *B. bigemina*.

## Conclusions

In summary, a *B. bigemina* transient transfection system able to transfer exogenous DNA into both *B. bigemina* and *B. bovis* parasites was developed. The locus of the *B. bigemina* Puerto Rico strain *elongation factor-1α* was identified, characterized and the transcriptional activity of *ef-1α* promoters tested. This study demonstrates the existence of two distinct promoters with interchangeable homologous and heterologous promoter function in *B. bigemina* and *B. bovis*. The observation of interspecies promoter activity, described for the first time in *Babesia* species, is an important step for future stable transfection construct design and for the production of vaccines based on transfected parasites for *B. bigemina* and *B. bovis*.

## Additional file

Additional file 1:
**Figure S1. a** In vitro growth curve of *B. bigemina* parasites. The Y axis represents the PPE and X axis represents the different times points of measurement in hours. The transfected plasmid constructs are represented in lines Ig-A (blue diamond), Ig-A Rev (red square), Ig-B (green triangle), Ig-B Rev (purple x), 4-35-ef-luc (light blue asterisk), Pr- (orange circle) pBluescript (dark blue bar). **b** In vitro growth curve of *B. bovis* parasites. The Y axis represents the PPE and X axis represents the different times points of measurement in hours. The transfected construct plasmid are represented in lines Ig-A (blue diamond), Ig-A Rev (red square), Ig-B (green triangle), Ig-B Rev (purple x), 4-35-ef-luc (light blue asterisk), Pr- (orange circle), pBluescript (pBS) (dark blue bar). Black bars represent standard deviation. ANOVA was used as statistical method of analysis. The assay was performed in triplicate. (TIF 517 kb)

## Abbreviations

bp: Base pair
ccp-3: Limulus coagulation factor C domain protein-3
*ef-1α*: Elongation factor 1-alpha
gDNA: genomic DNA
IG: Intergenic
IR: Inverted repeats
iRBC: Infected red blood cells
KO: Knockout
luc: Luciferase
msa-1: Merozoites surface protein-1
orfs: Open reading frames
PCR: Polymerase chain reaction
pDNA: plasmid DNA
PPE: Percentage of parasitized erythrocytes
qPCR: quantitative real-time PCR
RACE: Rapid amplification of cDNA ends
RLU: Relative luciferase unit
SD: Standard deviation
TBDs: Tick and tick-borne diseases
UTR: Untranslated region
