# Supplementary figures and images for: Identification of interchangeable cross-species function of elongation factor-1 alpha promoters in Babesia bigemina and Babesia bovis
Source: Parasit Vectors. 2016 Nov 11;9:576. doi: 10.1186/s13071-016-1859-9 (PMC5106780; doi:10.1186/s13071-016-1859-9)

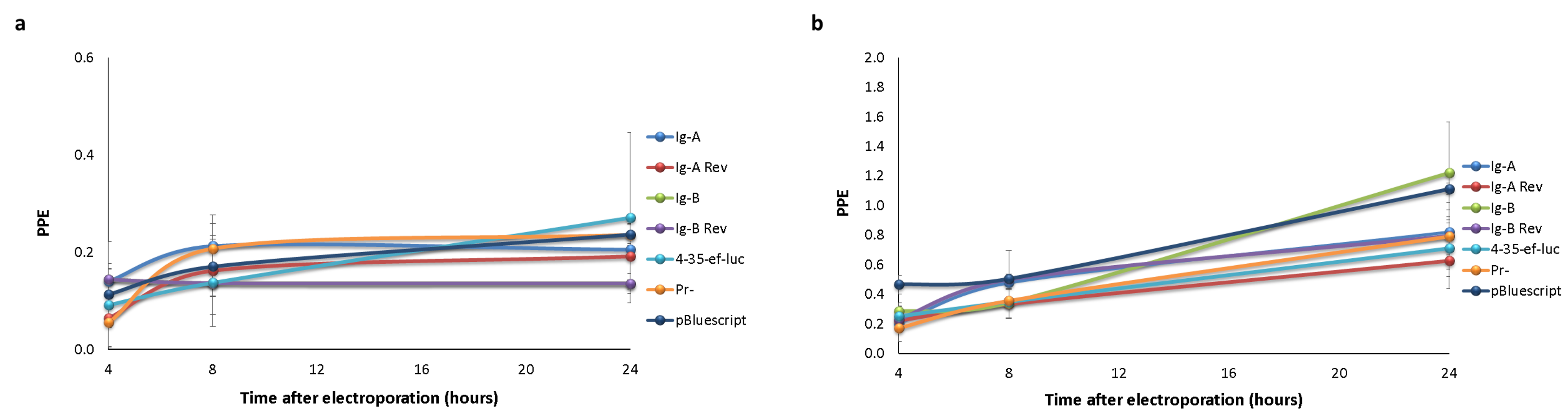

Supplement: Additional file 1: — Figure S1. a In vitro growth curve of B. bigemina parasites. The Y axis represents the PPE and X axis represents the different times points of measurement in hours. The transfected plasmid constructs are represented in lines Ig-A (blue diamond), Ig-A Rev (red square), Ig-B (green triangle), Ig-B Rev (purple x), 4-35-ef-luc (light blue asterisk), Pr- (orange circle) pBluescript (dark blue bar). b In vitro growth curve of B. bovis parasites. The Y axis represents the PPE and X axis represents the different times points of measurement in hours. The transfected construct plasmid are represented in lines Ig-A (blue diamond), Ig-A Rev (red square), Ig-B (green triangle), Ig-B Rev (purple x), 4-35-ef-luc (light blue asterisk), Pr- (orange circle), pBluescript (pBS) (dark blue bar). Black bars represent standard deviation. ANOVA was used as statistical method of analysis. The assay was performed in triplicate. (TIF 517 kb) [file 13071_2016_1859_MOESM1_ESM.tif]
